# Association of the Apolipoprotein E 2 Allele with Concurrent Occurrence of Endometrial Hyperplasia and Endometrial Carcinoma

**DOI:** 10.1155/2015/593658

**Published:** 2015-02-09

**Authors:** Tatiana I. Ivanova, Ludmila I. Krikunova, Nikolay I. Ryabchenko, Liana S. Mkrtchyan, Vera A. Khorokhorina, Lyubov E. Salnikova

**Affiliations:** ^1^A. Cyb Scientific Centre of Radiology of the Hertsen Federal Medical Research Centre of the Ministry of Health of the Russian Federation, 10 Zhukov Street, Obninsk, Kaluga Region 249036, Russia; ^2^Federal State Budget Institution of Sciences Institute of Gene Biology, Russian Academy of Sciences, 34/5 Vavilova Street, Moscow 119334, Russia; ^3^Federal State Budget Institution of Sciences N.I. Vavilov Institute of General Genetics, Russian Academy of Sciences, 3 Gubkin Street, Moscow 117971, Russia; ^4^Federal Research Center of Pediatric Hematology, Oncology and Immunology Named after Dmitry Rogachev, The Russian Ministry of Health and Social Development, 1 Samora Machel Street, Moscow 117198, Russia

## Abstract

Genes encoding proteins with antioxidant properties may influence susceptibility to endometrial hyperplasia (EH) and endometrial carcinoma (ECa). Patients with EH (*n* = 89), EH concurrent with ECa (*n* = 76), ECa (*n* = 186), and healthy controls (*n* = 1110) were genotyped for five polymorphic variants in the genes involved in metabolism of lipoproteins (*APOE* Cys112Arg and Arg158Cys), iron (*HFE* Cys282Tyr and His63Asp), and catecholamines (*COMT* Val158Met). Patients and controls were matched by ethnicity (all Caucasians), age, body mass index (BMI), and incidence of hypertension and diabetes. The frequency of the *APOE* E 2 allele (158Cys) was higher in patients with EH + ECa than in controls (*P* = 0.0012, *P*_Bonferroni_ = 0.018, OR = 2.58, 95% CI 1.49–4.45). The *APOE* E 4 allele (112Arg) was more frequently found in patients with EH than in controls and *HFE* minor allele G (63Asp) had a protective effect in the ECa group, though these results appeared to be nonsignificant after correction for multiple comparisons. The results of the study indicate that E 2 allele might be associated with concurrent occurrence of EH and ECa.

## 1. Introduction

Cancer of the endometrium is the most common gynecologic malignancy in developed countries and the second most common in developing countries [1]. Most cases (75–85%) of endometrial carcinoma occur in the sixth and seventh decades of life, and 95% occur in patients over 40 years of age [2, 3]. In general, endometrial carcinoma tends to have a favorable prognosis, but it is still a life-threatening disease. A number of deaths from endometrial cancer in the United States doubled between 1988 and 1998, probably due to an increase in life expectancy and high prevalence of obesity, diabetes, and hypertension, which predispose to this disease [4]. Amongst all cancers, endometrial cancer has the strongest association with obesity [5], a state characterized by chronic oxidative stress [6]. Adipocytes and inflammatory cells from visceral adipose tissue depots produce adipokines and cytokines which promote tumor development. Adipocyte mediated conversion of androgens to estrogen can add high risk to the development of endometrial cancer [7]. Associated with aging oxidative stress occurs in many tissues whose sensitivity to reactive oxygen species (ROS) appears to arise with increasing age [8, 9].

Not all females exposed to the same risk factors will develop an endometrial cancer. Although the most common types of endometrial cancer (e.g., endometrioid carcinoma) often develop from the endometrial hyperplasia, the latter will not necessarily progress to malignancy. An individual's susceptibility to disease development and progression is influenced by genetics. A few case-control studies have investigated the correlation of single nucleotide polymorphisms (SNPs) with hyperplasia of endometrium alone or in combination with endometrial cancer. It was shown that variant alleles of the* CYP1A1* and* CYP17* genes might be associated with endometrial hyperplasia (EH) and endometrial adenocarcinoma (ECa) susceptibility [10, 11]. Morosova et al. found that* MMP1* variant may be a risk marker of myo- and endometrial hyperplasia [12]. The results of these assays suggest that identification of genetic risk factors may provide a screening tool to reveal individuals at increased risk of development of hyperplasia and its progression to endometrial cancer. The present study examines the role of functional genetic polymorphisms in the genes encoding enzymes with the antioxidant properties being involved in metabolism of lipoproteins (*APOE*), iron (*HFE*), and catecholamines (*COMT*) in patients with EH, EH concurrent with ECa, and ECa without EH.

## 2. Materials and Methods

### 2.1. Patients and Controls

From August 2006 to August 2014 we selected a group of hospitalized and ambulatory patients with EH, ECa, and controls at A. Cyb Scientific Centre of Radiology of the Hertsen Federal Medical Research Centre of the Ministry of Health of the Russian Federation. A total of 1461 females from the European region of Russian Federation were recruited in the study. The study group consisted of 89 patients with histologically diagnosed simple EH (according to the endometrial intraepithelial neoplasia (EIN) nomenclature; [13]), 76 patients with ECa and EH, and 186 patients with ECa without hyperplasia. Exclusion criteria for the group under study consisted of age less than 18 years, lack of written informed consent, and previous history of any type of cancer. Females with EIN, adenomyosis, endometriosis, and types of cancer other than ECa (e.g., uterine sarcoma) were excluded. The patients were diagnosed as having ECa and EH by fractional endometrial biopsy. Total hysterectomy, bilateral pelvic and para-aortic lymphadenectomy, and omentectomy were performed in ECa patients. All cases were microscopically verified. The tumors were staged according to the International Federation of Gynecology and Obstetrics (FIGO) classification [14]. The study protocol was approved by the Ethics Committee of A. Cyb Scientific Centre of Radiology of the Hertsen Federal Medical Research Centre of the Ministry of Health of the Russian Federation and adhered to the tenets of the Declaration of Helsinki.

### 2.2. Genotyping

DNA was extracted from 500 *μ*L of whole blood using Wizard Genomic DNA Purification Kit (Promega, Madison, WI, USA). Genotypes were determined by a PCR-based restriction fragment length polymorphism (RFLP) technique. The 310 bp fragment of the* APOE* gene exon 4 which contains both polymorphic codons (Cys282Tyr and His63Asp) was amplified using primers F 5′-GAGACGCGGGCACGGCTGTCC and R 5′-GCACGCGGCCCTGTTCCACC. The 389 bp fragment of the* HFE* gene including codon Cys282Tyr was amplified with primers F 5′-TGGCAAGGGTAAACAGATCC and R 5′-CTCAGGCACTCCTCTCAACC; the 208 bp fragment of the* HFE* gene containing codon His63Asp was amplified using primers F 5′-ACATGGTTAAGGCCTGTTGC and R 5′-GCCACATCTGGCTTGAAATT. Primers used to amplify a 169 bp of the* COMT* gene containing codon Val158Met were F 5′-ACTGTGGCTACTCAGCTGTG and R 5′-CCTTTTTCCAGGTCTGACAA [15]. The fragments were amplified in 25 *μ*L reaction volume containing 0,625 U of HotStarTag polymerase (Qiagen, Valencia, USA), 0,15 mM dNTPs, 0,2 *μ*M primers, and 50 ng of genomic DNA. PCR-reactions were carried out as follows: at first 15 min denaturing at 95°C and then 30 amplification cycles (30 s at 94°C, 30 s at 67°C, 1 min at 72°C) for* APOE*; 30 cycles (30 s at 94°C, 1 min at 60°C, 30 s at 72°C) for* HFE*; and 40 cycles (30 s at 94°C, 1 min at 57°C, 30 s at 72°C) for* COMT*. A final extension of 10 min at 72°C was then applied for all reactions. Amplified fragments were digested with either Rsa I (Promega, Madison, WI, USA) or BclI (SIGMA, Saint Louis, MO, USA) for detection of the Cys282Tyr or His63Asp of the* HFE* gene; Cfo (Promega, Madison, WI, USA) for the* APOE *genotype determination and Hsp92II (Promega, Madison, WI, USA) for the Val158Met of the* COMT* gene. Digests were performed according to the manufacturer's instructions. PCR products and digested fragments were run on 8% polyacrylamide gels, stained with ethidium bromide, and visualized by UV. For quality controls, 10% of random samples of DNA were genotyped twice and the results were concordant for all duplicated sets.

### 2.3. Statistical Analysis

Deviation from Hardy-Weinberg equilibrium (HWE) was assessed by *χ* ^2^ analysis. Two-tailed Fisher's exact test (implemented in the WINPEPI computer programs; module A in COMPARE2 package [16]) was performed to evaluate differences between clinical and tumor characteristics of patients. The distributions of genotypes and haplotypes in cases and controls were compared using a logistic regression analysis, implemented in SNPStats package [17]. SNPStats is a free web-based tool for genetic association studies. The association with disease is modeled with unconditional logistic regression. In the analysis of the SNPs in relation to the response, SNPStats provides odds ratios (ORs), the confidence interval (CI), the *P* values for multiple inheritance models (dominant (var/var and wt/var versus wt/wt), recessive (var/var versus wt/var and wt/wt), overdominant (wt/var versus wt/wt and var/var), codominant (wt/wt versus wt/wt; wt/var versus wt/var; var/var versus var/var), and additive (wt allele versus var allele)), and the Akaike Information Criterion (AIC) indicating the best genetic model for each SNP. In multivariate models, quantitative or categorical variables may be additionally included in the regression models to be considered as potential confounders. Multivariate models predict outcomes that are affected by more than one variable. In multivariate models, we adjusted for age, body mass index (BMI), hypertension, diabetes, smoking habits, and using of hormone replacement therapy (HRT). For the regression models we present OR for the minor allele. The best genetic model was selected using the AIC value. The lowest AIC value was considered the best-fitting model for the fitted variant. For genotypes with minor allele frequencies <10% only dominant and additive genetic models were considered.

To exclude the false-positive associations, we used Bonferroni correction for multiple comparisons. To perform correction for multiplicity we divided the critical* P *value by the number of comparisons being made (5 SNPs and three groups of cases; 15 comparisons). The cut-off* P* value was 0.05/15 = 0.0033. When we considered* APOE* reference genotype 3/3 in comparison with all other* APOE* genotypes, significant *P* value was set at 0.05/12 = 0.0041.

WINPEPI test power and sample size calculators (in COMPARE2 package) were used to evaluate type II error [16]. The statistical power of association studies strongly depends on the sample size, genetic model, and genotype frequencies [18]. An effect size of 2.0 and higher is thought to be clinically meaningful [19]. To detect OR = 2.00 for minor allele frequencies 0.10 and 0.25 in our sample size, the power ranges from 41.19% to 81.24% and from 73.10% to 97.88%, respectively. Post hoc power calculations showed that, for a main effect, statistical power was 88.43% (EH + ECa group,* APOE* rs7412, OR = 2.58).

## 3. Results

### 3.1. Characteristics of the Study Population

Clinicodemographic characteristics of the group under study are presented in Table 1. Patients and controls were matched by ethnicity (all Caucasians), age, and BMI. There were no differences between the study groups in relation to hypertension and diabetes incidence. With the exception of the group ECa, the frequencies of smoking (ever smoking versus never smoking) did not differ between patients and controls. Striking contrast was found between groups under study in relation to ever use of HRT. In controls, EH, and EH + ECa groups, 20%, 18%, and 18% of females used HRT, while in the ECa group, only 10% of females took HRT (*P* = 0.001).

Tumor characteristics of patients are given in Table 2. EH + ECa patients had a more favorable stage and grade than ECa patients. For example, 92.11% EH + ECa patients were FIGO I-II stages and 81.58% G1-2 grades (well and moderately differentiated). In the ECa group, FIGO I-II stages and G1-2 grades had 86.56% and 77.96% of subjects, respectively.

### 3.2. Genetic Associations of Selected Candidate Genes in EH, EH + ECa, and ECa Studies

The genotype distributions of the* COMT *(Val158Met),* HFE* (Cys282Tyr and His63Asp), and* APOE* (Cys112Arg and Arg158Cys) polymorphisms between cases and healthy controls are shown in Table 3. With the exception for the* HFE* rs1799945 in the ECa group, the genotype frequencies of the polymorphisms in controls and cases were consistent with the Hardy-Weinberg equilibrium distribution. A significant increase in the frequency of the E 2 allele (*APOE* rs7412-T, 158Cys) was observed in patients with EH + ECa compared with controls in both crude (*P* = 2.6 × 10^−5^, *P*_Bonferroni_ = 3.9 × 10^−4^, OR = 3.12, 95% CI 1.89–5.17) and multivariate analyses (*P* = 0.0012, *P*_Bonferroni_ = 0.018, OR = 2.58, 95% CI 1.49–4.45). Though nonsignificant after Bonferroni correction for multiple comparisons, the E 4 allele (*APOE* rs429358-C, 112Arg) was more frequently found in patients with EH than in controls and* HFE* rs1799945 minor allele G had a protective effect in the ECa group.

To further exploit the role of* APOE* E 2 allele in concurrent occurrence of EH and ECa, we performed an additional study of clinicodemographic characteristics in relation to different* APOE* alleles. In patients with EH + ECa, E 2 allele was not associated with BMI, hypertension, diabetes, smoking habits, and HRT (Table 4) or with different tumor grades (G1 versus G2-3) (Table 5).

Next, we employed the distribution of the* APOE* reference E 3/E 3 (112Cys-158Arg/112Cys-158Arg) and variant genotypes in controls and cases (Table 6). The effects appeared to be less pronounced: variant genotypes (all together) were associated with EH (*P* = 0.038, *P*_Bonferroni_ > 0.05, OR = 1.60, 95% CI 1.04–2.47) and EH + ECa (*P* = 0.004, *P*_Bonferroni_ = 0.048, OR = 1.98, 95% CI 1.24–3.15).

In the overall cancer group (EH + ECa and ECa),* APOE *E 2 allele was more frequent among patients compared with controls, but the results were nonsignificant after multiple testing corrections (*P* = 0.036, *P*_Bonferroni_ > 0.05, OR = 1.54, 95% CI 1.03–2.30).

Haplotype analysis of the* HFE* gene polymorphisms was undertaken and the estimated haplotype frequencies are presented in Table 7. No significant differences were observed between controls and patients in any group in regard to haplotype frequencies. Haplotype comprising both minor alleles rs1800562-A/rs1799945-G was not found in our sample.

## 4. Discussion

In this study of five polymorphisms in antioxidant-related genes (two each in* APOE* and* HFE*, one in* COMT*), we found that* APOE* E 2 allele (158Cys) was associated with a concurrent occurrence of EH and ECa in a cohort of Caucasian patients from the European region of Russian Federation. The* APOE* E 4 allele (112Arg) was more frequently found in patients with EH than in controls and* HFE* minor allele G (63Asp) had a protective effect in the ECa group. Only the effect of the* APOE* E 2 allele in EH + ECa patients appeared to be significant after correction for multiple testing procedures.

Endometrial carcinoma may arise and evolve through divergent pathways and different precursor lesions. Based on histopathology and molecular alterations, endometrial cancers are divided into two major pathogenetic variants [20]. The first pathogenetic variant comprises low-grade (G1-2) adenocarcinomas that are typically diagnosed early, are usually estrogen dependent, and have a favorable prognosis. The pathway of this variant of adenocarcinoma includes several steps: simple hyperplasia, atypical hyperplasia, and endometrial carcinoma [20]. The second pathogenetic variant comprises adenocarcinomas that are not hormone dependent and are usually grade 3 (G3) tumors that are associated with early spread and worse prognosis. It is assumed that the second pathogenetic variant of adenocarcinomas can arise from dedifferentiation of a preexisting first pathogenetic variant of cancer [21]. Though considered benign, simple endometrial hyperplasia is followed by ECa in 19% of the cases; complex hyperplasia with atypia is recognized as an early malignant lesion and occurs concurrently with ECa in 39% of the cases [22]. In general, tumor features of our groups under study are in line with the above characteristics. There are more cases with lower grades and FIGO stages in the group with the concurrent occurrence of hyperplasia and adenocarcinoma than in the group with adenocarcinoma only. Compared to the ECa cases, females with EH + ECa were the more frequent users of HRT, which may be associated with the increased risk of estrogen-related endometrial cancer.

APOE is one of the major plasma lipoproteins with antioxidant, anti-inflammatory, and antiatherogenic properties [23]. APOE is presented in multiple normal and cancer tissues, including normal human endometrium [24], EH, and ECa [25, 26]. It is upregulated in many types of human cancer: ovarian [27, 28], pancreatic [29], prostate [30], gastric [31, 32], and anaplastic thyroid cancer [33] and glioblastoma [34]. In gastric cancer, anaplastic thyroid carcinoma, and poorly differentiated endometrial adenocarcinoma, APOE overexpression was associated with advanced grade and stage or more aggressive low differentiated tumors [26, 31, 33]. Upregulation of APOE precursor was observed also in simple endometrial hyperplasia [35].* In vivo* experiments in hamsters have shown that overexpression of Apoe may play significant role in the malignant transformation of oral mucosa precancerous lesions to squamous cell carcinoma [36]. Increased APOE expression in hyperplasia and cancer are not fully understood. APOE may be involved in signal transduction and lipid transport essential for proliferation and survival of tumor cells or may potentiate tumor proliferation and survival maintaining a specific microenvironment [28]. Apolipoprotein E is also produced in macrophages [37]. Tumor-associated macrophages (TAMs) have been shown to relate to vascular space invasion and myometrial invasion in ECa [38]. TAMs are also involved in progression of precancerous endometrial lesions in ECa [39].

APOE isoforms differ in amino acid residues at positions 112 and 158. E 3, the most common isoform, contains cysteine and arginine at these codons; E 2 has two cysteines and E 4 two arginines. E 2 is characterized by a 50–100-fold weaker binding affinity of the protein for cell surface LDL receptors that leads to type III hyperlipoproteinaemia, that is, high circulating triglycerides (TAG) levels. It is assumed that E 2 has the greatest stability and least formation of intermediate metabolites. Amino acid substitution resulting in E 4 isoform effects chemical and thermal stability through conformation changes and reveals better lipid binding activity [40]. Isoforms exhibit the differential lipoprotein binding preferences. E 2 and E 3 prefer interaction with small lipoproteins such as HDL, while E 4 more frequently binds to larger lipid-rich lipoproteins (very-low-density lipoprotein, VLDL, and low-density lipoprotein, LDL). E 2 has a potential beneficial effect on lipid profile, especially total cholesterol (TC) and low-density lipoprotein-cholesterol (LDL-C) levels; however, E 2 carriers have higher BMI, waist circumference, homeostasis model insulin resistance index, triglyceride/high density lipoprotein-cholesterol ratio, and increased risk of metabolic syndrome. APOE E 4 is associated with increased serum triglyceride (TG), TC and LDL-C levels [41], and the diseases with known or proposed association with oxidative stress and a proinflammatory status, for example, coronary heart disease and stroke [42], Alzheimer's disease [43], and psoriasis [44].

Oxidative stress is recognized to be involved in many disorders of the female reproductive system. Alterations of redox status have been shown in blood of patients with EH and ECa [45]. Tissues undergoing oncogenic transformation may acquire additional energy source with an increased synthesis of endogenous fatty acids [46]. The fatty acid metabolism may promote oxidative stress in hyperplastic tissue [47, 48]. Antioxidant and anti-inflammatory APOE activity is genotype-dependent (E 2 > E 3 > E 4) [49]. Different mechanisms of the implementation of APOE antioxidant function are discussed: metal sequestration [50, 51], free radical scavenging activity of the region rich in positively charged amino acids [52], and binding and detoxifying of 4-hydroxynonenal (HNE) [53].

E 2 allele has never been studied in relation to endometrial cancer, though syndromes and biochemical characteristics associated with E 2 allele are known to predispose to endometrial cancer. Increased endometrial cancer risk is observed in patients with insulin resistance/metabolic syndrome, hypertriglyceridemia, and obesity [54–57]. The adipose tissue is a very important source of endogenous estrogens in postmenopausal women. Estrogens produced in adipose tissue exhibit mitogenic activity on endometrial cells that leads to an increased risk of endometrial cancer [54]. Estrogens may induce oxidative stress by different mechanisms [58] and oxidative stress, in turn, leads to the excessive formation of semiquinones and quinones from catechol estrogens. Oxidation of catechol estrogens to semiquinones and then to quinones is a pathway that generates reactive oxygen species (ROS) such as hydroxyl radicals (^∙^OH) that can cause DNA damage and initiate cancer [59]. Cancer cells increase their production of mitochondrial ROS to further stimulate neoplastic transformation [60]. Oxidative stress and free radicals are important activators for apoptosis [61], and if cancer is already present, free radicals are important for apoptosis signals [62]. Tumor cells proliferate faster when oxidative stress is suppressed. It was shown that antioxidants may stimulate the growth of early tumors or precancerous lesions in high-risk populations [63]. In the current study, we observed that E 2 allele with the highest antioxidant activity was more frequent in patients with concurrent occurrence of EH and ECa. These results support the increasing experimental data on the protective effects of the oxidative stress against further growth and malignization of precancerous lesions.

In the present assay we found that* APOE* E 4 allele was associated with EH and observed a protective effect of* HFE *rs429358 allele G against ECa. Both results failed to pass Bonferroni's correction for multiple statistical tests and may be only taken into account for future research directions. To the best of our knowledge, data about the role of E 4 allele in hyperplasia and endometrial cancer are absent. Among other hormone-dependent cancers, it has been shown that E 4 allele is a low-penetrant risk factor for breast cancer [64] and prostate cancer [65]. In large samples of longitudinally followed populations, shorter life expectancy was assumed in females due to non-sex-specific cancers, associated with E 4 allele [66].* HFE* (high iron) is a major histocompatibility complex (MHC) class I-like gene. HFE protein regulates iron uptake into cells and is predominantly expressed in tissues involved in iron storage, such as hepatocytes and macrophages. Most individuals (80–90%) affected by hemochromatosis (iron overload) are homozygous for the Cys282Tyr substitution.* HFE* variant His63Asp has a very low penetrance and mild expressivity for iron overload [67].* HFE* Cys282Tyr variant allele is associated with breast cancer [68], colorectal cancer [69], and hepatocellular carcinoma [70]. A protective role of this variant was found against chronic myeloproliferative disease [71].* HFE* His63Asp variant allele was more frequent in gastric cancer [72].

Our study has both limitations and strengths. The main limitations are the relatively low number of patients and unavailable data on the receptor status of the ECa. The important strengths are as follows. Our controls and all groups of cases have the same clinical and demographic characteristics. We selected a large control group and allele frequencies were very close to those found in other Caucasian populations. Statistical analyses included all main covariates important in regard to EH and ECa risk.

In summary, the role of functional SNPs in the* COMT*,* APOE, *and* HFE* genes in EH and ECa was elucidated in Caucasians from Russian Federation. Current data provide, for the first time, strong evidence that E 2 allele of the* APOE* gene is associated with concurrent occurrence of EH and ECa. These results are in line with known associations of this allele with diseases or traits predisposing to ECa, such as obesity, metabolic syndrome, and hypertriglyceridemia. Further studies with larger sample sizes and other populations are required to replicate our results.

## Figures and Tables

**Table 1:** Clinical characteristics in patients and controls.

Characteristics	Controls (*N* = 1110) *N* (%) Mean ± SD	EH (*N* = 89)		EH + ECa (*N* = 76)		ECa (*N* = 186)	
		*N* (%) Mean ± SD	*P*	*N* (%) Mean ± SD	*P*	*N* (%) Mean ± SD	*P*
Age (years)	59.96 ± 12.17	58.69 ± 10.20	0.32	57.90 ± 10.61	0.14	58.60 ± 9.62	0.13
BMI (kg/m^2^)	29.44 ± 5.30	28.58 ± 5.79	0.14	30.09 ± 9.00	0.33	30.04 ± 7.25	0.18
Smoking	122 (10.99)	6 (6.74)	0.28	4 (5.26)	0.17	11 (5.91)	**0.036**
Hypertension	426 (38.38)	35 (39.33)	0.91	34 (44.74)	0.28	80 (43.01)	0.26
Diabetes	39 (3.51)	1 (1.12)	0.36	6 (7.89)	0.062	12 (6.45)	0.066
HRT	221 (19.91)	16 (17.98)	0.78	14 (18.42)	0.88	19 (10.22)	**0.001**

Significant results are in bold.

**Table 2:** Tumor characteristics of patients with ECa.

Characteristics	EH + ECa (*n* = 76)	ECa (*N* = 186)	*P*
	*N* (%)	*N* (%)	
Histopathologic grades			
G1 (well differentiated)	34 (44.74)	48 (25.81)	**0.003**
G2 (moderately differentiated)	28 (36.84)	97 (52.15)	**0.029**
G3 (poorly or undifferentiated)	7 (9.21)	8 (4.30)	0.14
Gx (N/A)	7 (9.21)	33 (17.74)	0.091

Histopathologic types (WHO/ISGP)			
Endometrioid adenocarcinoma	71 (93.42)	150 (80.65)	**0.009**
Clear cell adenocarcinoma	4 (5.26)	1 (0.54)	**0.026**
Adenosquamous carcinoma	0	2 (1.08)	—
Papillary serous adenocarcinoma	1 (1.32)	8 (4.30)	0.45
x (N/A)	0	25 (13.44)	—

FIGO stages			
I	67 (88.16)	131 (70.43)	**0.002**
II	3 (3.95)	30 (16.13)	**0.007**
III	2 (2.63)	13 (6.99)	0.24
IV	2 (2.63)	3 (1.61)	0.63
X (N/A)	2 (2.63)	9 (4.84)	0.52

N/A: not available. Significant results are in bold.

**Table 3:** The distribution of genotypes in control subjects and patients with EH, EH + ECa, and ECa.

Genes and genotypes		Controls number (%)	EH		*P* mult OR (95% CI)	EH + ECa		*P* mult OR (95% CI)	ECa		*P* mult OR (95% CI)
			Number (%)	*P* crude OR (95% CI)		Number (%)	*P* crude OR (95% CI)		Number (%)	*P* crude OR (95% CI)	
*COMT* rs4680 G1947A Val158Met		HWE	HWE			HWE			HWE		
		*P* = 0.18	*P* = 0.39			*P* = 0.64			*P* = 0.54		
		*n* = 1074	*n* = 86			*n* = 73			*n* = 170		
	A/A	273 (25.4)	26 (30.2)	0.33 (dom)	0.30 (dom)	17 (23.3)	0.68 (dom)	0.68 (dom)	44 (25.9)	0.75 (add)	0.74 (add)
	G/A	559 (52.0)	39 (45.4)	0.79	0.77	39 (53.4)	1.12	1.13	90 (52.9)	0.96	0.96
	G/G	242 (22.5)	21 (24.4)	(0.49–1.27)	(0.47–1.25)	17 (23.3)	(0.64–1.97)	(0.63–2.04)	36 (21.2)	(0.76–1.22)	(0.73–1.25)

*HFE* rs1800562 G845A Cys282Tyr		HWE	HWE			HWE			HWE		
		*P* = 0.58	*P* = 1.00			*P* = 1.00			*P* = 0.20		
		*n* = 1106	*n* = 89			*n* = 76			*n* = 186		
	G/G	1045 (94.5)	84 (94.4)	0.97 (dom)	0.98 (dom)	71 (93.4)	0.70 (dom)	0.91 (dom)	174 (93.5)	0.48 (add)	0.56 (add)
	G/A	60 (5.4)	5 (5.6)	1.02	1.01	5 (6.6)	1.21	1.06	11 (5.9)	1.25	1.22
	A/A	1 (0.1)	0 (0.0)	(0.40–2.61)	(0.39–2.60)	0 (0.0)	(0.48–3.10)	(0.39–2.89)	1 (0.5)	(0.69–2.27)	(0.63–2.35)

*HFE* rs1799945 C187G His63Asp		HWE	HWE			HWE			**HWE**		
		*P* = 0.57	*P* = 0.069			*P* = 0.59			*P* = 0.0058		
		*n* = 1105	*n* = 89			*n* = 76			*n* = 186		
	C/C	783 (70.9)	62 (69.7)	0.32 (add)	0.36 (add)	57 (75.0)	0.28 (add)	0.32 (add)	149 (80.1)	**0.015** (**dom**)^§^	**0.024** (**dom**)^§§^
	C/G	298 (27.0)	21 (23.6)	1.23	1.21	19 (25.0)	0.76	0.77	30 (16.1)	**0.60**	**0.62**
	G/G	24 (2.2)	6 (6.7)	(0.83–1.82)	(0.81–1.80)	0 (0.0)	(0.46–1.26)	(0.46–1.30)	7 (3.8)	(**0.41–0.89**)	(**0.41–0.95**)

*APOE* rs7412 C526T Arg158Cys E2 allele (158Cys)		HWE	HWE			HWE			HWE		
		*P* = 0.37	*P* = 1.00			*P* = 0.44			*P* = 1.00		
		*n* = 1065	*n* = 89			*n* = 76			*n* = 177		
	C/C	913 (85.7)	73 (82.0)	0.35 (dom)	0.36 (dom)	50 (65.8)	2.6 × 10^−5^ (**dom**)^#^	**0.0012** (**dom**)^##^	149 (84.2)	0.59 (dom)	0.64 (dom)
	C/T	144 (13.5)	16 (18.0)	1.32	1.32	25 (32.9)	**3.12**	**2.58**	27 (15.2)	1.13	1.13
	T/T	8 (0.8)	0 (0.0)	(0.75–2.32)	(0.74–2.33)	1 (1.3)	(**1.89–5.17**)	(**1.49–4.45**)	1 (0.6)	(0.73–1.75)	(0.68–1.86)

*APOE* rs429358 T388C Cys112Arg E4 allele (112 Arg)		HWE	HWE			HWE			HWE		
		*P* = 0.20	*P* = 0.45			*P* = 1.00			*P* = 1.00		
		*n* = 1065	*n* = 89			*n* = 76			*n* = 177		
	T/T	827 (77.7)	60 (67.4)	**0.034** (**dom**)^*^	**0.026** (**dom**)^**^	57 (75.0)	0.60 (dom)	0.38 (dom)	140 (79.1)	0.56 (add)	0.69 (add)
	T/C	218 (20.5)	28 (31.5)	**1.68**	**1.73**	18 (23.7)	1.16	1.29	35 (19.8)	0.90	0.92
	C/C	20 (1.9)	1 (1.1)	(**1.05–2.68**)	(**1.08–2.77**)	1 (1.3)	(0.68–1.99)	(0.73–2.28)	2 (1.1)	(0.63–1.28)	(0.62–1.37)

Rs number is a reference SNP ID number in The Single Nucleotide Polymorphism database, dbSNP.

The choice of each genetic model was based on AIC value. The genetic model: rec, recessive; dom, dominant; add, additive. OR, odds ratio; CI, confidence interval; mult, multivariate.

In multivariate analysis we adjusted for age, BMI, hypertension, diabetes, smoking habits, and using of HRT.

Significant results are in bold.

Bonferroni adjusted *P* values for significant results ^*^ *P*_Bonferroni_ = 0.51; ^**^ *P*_Bonferroni_ = 0.39; ^#^ *P*_Bonferroni_ = 3.9 × 10^−4^; ^##^ *P*_Bonferroni_ = 0.018; ^§^ *P*_Bonferroni_ = 0.23; ^§§^ *P*_Bonferroni_ = 0.36.

**Table 4:** The distributions of BMI, hypertension, diabetes, smoking habits, and HRT in patients with EH + ECa with different *APOE* E2 genotypes.

Characteristics	C/C (*n* = 50) Number Mean ± SD	C/T-T/T (*n* = 26) Number Mean ± SD	*P* value
Age (years)	56.90 ± 11.11	59.82 ± 9.49	0.26
BMI (kg/m^2^)	30.56 ± 8.93	29.18 ± 9.14	0.53
Smoking	2	2	0.60
Hypertension	24	10	0.47
Diabetes	5	1	0.66
HRT	10	4	0.76

**Table 5:** The distribution of genotypes in patients with EH + ECa with different tumor grades.

Genes and genotypes		Grade I (G1) Number (%)	Grade II-III (G2-3) Number (%)	*P* crude OR (95% CI)	*P* mult OR (95% CI)
*COMT* rs4680 G1947A Val158Met		*n* = 34	*n* = 29		
	G/G	9 (26.47)	8 (27.6)	0.34 (rec)	0.34 (rec)
	G/A	19 (55.88)	14 (48.3)	1.78	1.81
	A/A	6 (17.65)	7 (24.1)	(0.54–5.90)	(0.54–6.10)

*HFE* rs1800562 G845A Cys282Tyr		*n* = 34	*n* = 31		
	G/G	32 (94.1)	28 (90.3)	0.57 (dom)	0.52 (dom)
	G/A	2 (5.9)	3 (9.7)	1.71	1.85
	A/A	0 (0.0)	0 (0.0)	(0.27–11.01)	(0.28–12.42)

*HFE* rs1799945 C187G His63Asp		*n* = 34	*n* = 31		
	C/C	28 (82.3)	21 (67.7)	0.17 (dom)	0.14 (dom)
	C/G	6 (17.6)	10 (32.3)	2.22	2.48
	G/G	0 (0.0)	0 (0.0)	(0.70–7.09)	(0.73–8.44)

*APOE* rs7412 C526T Arg158Cys (E2 allele)		*n* = 34	*n* = 31		
	C/C	19 (55.9)	23 (74.2)	0.12 (dom)	0.10 (dom)
	C/T	14 (41.2)	8 (25.8)	0.44	0.40
	T/T	1 (2.9)	0 (0.0)	(0.15–1.26)	(0.13–1.22)

*APOE* rs429358 T388C Cys112Arg (E4 allele)		*n* = 34	*n* = 31		
	T/T	28 (82.3)	20 (64.5)	0.10 (dom)	0.097 (dom)
	T/C	5 (14.7)	11 (35.5)	2.57	2.61
	C/C	1 (2.9)	0 (0.0)	(0.81–8.09)	(0.82–8.29)

The choice of each genetic model was based on AIC value. The genetic model: rec, recessive; dom, dominant. OR, odds ratio; CI, confidence interval; mult, multivariate.

In multivariate analysis we adjusted for age, BMI, hypertension, diabetes, smoking habits, and using of HRT.

**Table 6:** The distribution of the *APOE* E 3/E 3 reference and variant (including E 2 and E 4 alleles) genotypes in control subjects and patients with EH, EH + ECa, and ECa.

*APOE* genotypes		Controls number (%)	EH		EH + ECa		ECa	
			Number (%)	*P* mult OR (95% CI)	Number (%)	*P* mult OR (95% CI)	Number (%)	*P* mult OR (95% CI)
rs7412 + rs429358		*n* = 1065	*n* = 89	**0.038** ^*^	*n* = 76	**0.004** ^**^	*n* = 177	0.87
	E 3/E 3	695 (65.26)	48 (53.93)	**1.60**	37 (48.68)	**1.98**	114 (64.41)	1.04
	Other	370 (34.74)	41 (46.07)	** (1.04–2.47)**	39 (51.32)	** (1.24–3.15)**	63 (35.39)	(0.75–1.45)

*APOE* E 3 allele, 112Cys, and 158Arg; other, any genotypes with 112Arg and/or 158Cys.

OR, odds ratio; CI, confidence interval; mult, multivariate.

In multivariate analysis we adjusted for age, BMI, hypertension, diabetes, smoking habits, and using of HRT. Significant results are in bold.

Bonferroni adjusted *P* values ^*^ *P*_Bonferroni_ = 0.46; ^**^ *P*_Bonferroni_ = 0.048.

**Table 7:** Haplotype-based analysis of the *HFE* gene polymorphisms in EH, EH + ECa, and ECa.

*N*	rs1800562 G845A	rs1799945 C187G	Controls frequency (%)	EH (*n* = 1195)^*^		EH + ECa (*n* = 1182)^*^		EC (*n* = 1292)^*^	
				Frequency (%)	*P* mult OR (95% CI)	Frequency (%)	*P* mult OR (95% CI)	Frequency (%)	*P* mult OR (95% CI)
1	G	C	0.8154	0.7928	Reference	0.8421	Reference	0.8468	Reference
2	G	G	0.1566	0.1791	0.35 1.21 (0.81–1.80)	0.125	0.33 0.77 (0.45–1.30)	0.1183	0.15 0.77 (0.53–1.10)
3	A	C	0.028	0.0218	0.97 1.02 (0.40–2.58)	0.0329	0.98 0.99 (0.38–2.59)	0.0349	0.62 1.18 (0.61–2.28)

OR, odds ratio; CI, confidence interval; mult, multivariate.

^*^The number of subjects with both genotyped SNPs. Subjects with missing genotyping data for one of the SNPs were not included in the haplotype-based analysis.

In multivariate analysis we adjusted for age, BMI, hypertension, diabetes, smoking habits, and using of HRT.

## Abbreviations

AIC: Akaike's Information Criterion
APOE: Apolipoprotein E (protein)
*APOE*: Apolipoprotein E (gene)
BMI: Body mass index
COMT: Catechol-O-methyltransferase (protein)
*COMT*: Catechol-O-methyltransferase (gene)
CI: Confidence interval
ECa: Endometrial carcinoma (ECa)
EH: Endometrial hyperplasia
FIGO: The International Federation of Gynecology and Obstetrics
HFE: Hemochromatosis (protein)
*HFE*: Hemochromatosis (gene)
HRT: Hormone replacement therapy
OR: Odds ratio
ROS: Reactive oxygen species
SNP: Single nucleotide polymorphism.
